# S09-4: ‘Equality in the Field’

**DOI:** 10.1093/eurpub/ckae114.239

**Published:** 2024-09-26

**Authors:** Seamus Nugent

**Affiliations:** Sport Ireland, Dublin, Ireland; Kilkenny Local Sports Partnership, Ireland; LinkAge Consultancy

## Abstract

**Purpose:**

Sport participation and physical activity have a positive influence on the health of young people (Doull et al. 2018). However, in Irish schools 73% (n = 788) of LGBTQ+ students report feeling unsafe, while 77% have experienced verbal harassment based on their gender expression or sexual orientation (BeLonG, 2019). A consultation for strategy development by Kilkenny Recreation & Sports Partnership (KRSP) found that as an organisation it needs to improve its engagement with for the LGBTQ+ community and to develop practical support and training for sports organisations.

**Methods:**

A mixed methods study was conducted in November 2022 which used a transformative queer framework supported by gender affirmation theory. It focussed on LGBTQ+ groups of students, secondary school students and university students. In total 42 LGBTQ+ students attended focus groups and 41 completed a survey.

**Results:**

The built environment, especially changing rooms and toileting facilities, were deemed to be a major barrier to LGBTQ+ participation in sport and physical activity. Other findings highlighted the need for non-discriminatory policies, inclusive language and staff and volunteer training. Finally, it emerged that there is a need for an overall respectful, inclusive culture, one where bullying and harassment are not tolerated and where diverse gender expression and sexual identity is acceptable.

**Conclusion:**

It is critical that policymakers and service providers understand the factors which influence sport participation among the LGBTQ+ youth community. The findings underscore the importance of considering the unique barriers LGBTQ+ youth face during sport participation and the need for support/training to be provided for parents, coaches, educators, and sports providers. Considering that many of the study recommendations are low cost or no cost, the main challenge will be adopting appropriate practices which will support and guide intervention development.

KRSP are currently researching the awareness and uptake of training in LGBTQ+ theory and current / correct terminology by sports clubs and facilities in the Kilkenny/Carlow area. Following this they will develop, disseminate, and evaluate the use of a newly developed toolkit by sports clubs and sporting facilities in the study area.

**Support/Funding Source:**

Kilkenny Local Sports Partnership, LinkAge Consultancy

